# Changes in Corticospinal Circuits During Premovement Facilitation in Physiological Conditions

**DOI:** 10.3389/fnhum.2021.684013

**Published:** 2021-06-21

**Authors:** Giovanni Cirillo, Ilaria Antonella Di Vico, Mehran Emadi Andani, Francesca Morgante, Giovanna Sepe, Alessandro Tessitore, Matteo Bologna, Michele Tinazzi

**Affiliations:** ^1^Laboratory of Morphology of Neuronal Network, Division of Human Anatomy, Department of Mental, Physical Health and Preventive Medicine, University of Campania “Luigi Vanvitelli”, Naples, Italy; ^2^Movement Disorders Division, Neurology Unit, Department of Neurosciences, Biomedicine and Movement Sciences, University of Verona, Verona, Italy; ^3^Neurosciences Research Centre, Molecular and Clinical Sciences Research Institute, St George’s, University of London, London, United Kingdom; ^4^Department of Experimental and Clinical Medicine, University of Messina, Messina, Italy; ^5^Division of Neurology and Neurophysiopathology, Department of Medical and Surgical Sciences, University of Campania “Luigi Vanvitelli”, Naples, Italy; ^6^Department of Human Neurosciences, Sapienza University of Rome, Rome, Italy; ^7^IRCCS Neuromed, Pozzilli, Italy

**Keywords:** premovement facilitation, reaction time, transcranial magnetic stimulation, MEP, motor cortex

## Abstract

Changes in corticospinal excitability have been well documented in the preparatory period before movement, however, their mechanisms and physiological role have not been entirely elucidated. We aimed to investigate the functional changes of excitatory corticospinal circuits during a reaction time (RT) motor task (thumb abduction) in healthy subjects (HS). 26 HS received single pulse transcranial magnetic stimulation (TMS) over the primary motor cortex (M1). After a visual go signal, we calculated RT and delivered TMS at three intervals (50, 100, and 150 ms) within RT and before movement onset, recording motor evoked potentials (MEP) from the abductor pollicis brevis (APB) and the task-irrelevant abductor digiti minimi (ADM). We found that TMS increased MEP_APB_ amplitude when delivered at 150, 100, and 50 ms before movement onset, demonstrating the occurrence of premovement facilitation (PMF). MEP increase was greater at the shorter interval (MEP_50_) and restricted to APB (no significant effects were detected recording from ADM). We also reported time-dependent changes of the RT and a TMS side-dependent effect on MEP amplitude (greater on the dominant side). In conclusion, we here report changes of RT and side-dependent, selective and facilitatory effects on the MEP_APB_ amplitude when TMS is delivered before movement onset (PMF), supporting the role of excitatory corticospinal mechanisms at the basis of the selective PMF of the target muscle during the RT protocol.

## Introduction

Transcranial magnetic stimulation (TMS) has been widely used to investigate the function and plasticity inside the motor system and corticospinal pathway (Hallett, 2007; Huang et al., 2017; Moscatelli et al., 2021). When stimulating the primary motor cortex (M1), the magnetic field induces an electrical current throughout neuronal populations eliciting motor evoked potentials (MEP) from a target muscle recorded with surface electromyographic electrodes (Reis et al., 2008). Among neurophysiologic measures of cortical excitability that can be derived from single-pulse TMS, the MEP amplitude represents the result of the direct and indirect (*trans*-synaptic) excitation of a pool of corticospinal neurons beneath the TMS coil, which provides an immediate measure of cortical excitability at any given moment for any given condition (Pascual-Leone, 2000; Di Lazzaro, 2004; Monda et al., 2017).

When delivered in the preparation or execution phase of voluntary movements, single-pulse TMS can provide significant physiologic insights (Cirillo et al., 2017). For example, facilitation of MEP (i.e., amplitude increase) before a volitional movement in a target muscle (premovement facilitation, PMF), was demonstrated in normal adults (Rossini et al., 1988; Chen et al., 1998) and it likely reflects a building up of the corticospinal excitability in preparation for motor execution. PMF is defined as an increase of MEP amplitude/probability of MEP appearance after the go signal and before movement onset and begins approximately 80–100 ms before the electromyographic (EMG) onset (Hiraoka et al., 2010a). Evidence has demonstrated that it consists in a gradual increase of M1 neuronal activity above the threshold for discharging of spinal motor neurons (Morgante et al., 2011; Moscatelli et al., 2020).

To date, neurophysiological mechanisms of PMF with respect to the timing of TMS have not been yet fully clarified. Both excitatory and inhibitory circuits have been investigated (Reis et al., 2008) and modifications of corticospinal excitability have been well documented in the preparatory period before movement (Kaufman et al., 2014; Hannah et al., 2018), however, their physiological role has not been entirely elucidated (Rossini et al., 1988; Hoshiyama et al., 1996; Chen et al., 1998).

Since the time-dependent effects of TMS on MEP amplitude, changes of reaction time (RT) and the influence of hemispheric dominance have not been fully clarified, in this work we aimed to characterize the timing of PMF applying single-pulse TMS over M1 at three different intervals (50, 100, and 150 ms) before the EMG onset of the task movement (rapid thumb abduction) and we measured the amplitude of MEP, recording from the abductor pollicis brevis (APB), bilaterally, as index of excitatory circuits. We recorded MEP from both the APB (MEP_APB_) and the adjacent muscle abductor digiti minimi (ADM) (MEP_ADM_) to test the selectivity of our PMF protocol on the target muscle (APB), and to verify the possible occurrence of the surround inhibition (Sohn and Hallett, 2004), a relevant mechanism for selective movement execution.

## Materials and Methods

### Participants, Standard Protocol Approvals, Registrations, and Patient Consents

Twenty-six healthy subjects (HS) (13 male, 13 female, mean age 40.29 ± 2.9 years) naïve to the purpose of the experiment participated in the study. HS were all but two right-handed as demonstrated by the Edinburgh Handedness Inventory score (above 70) (Oldfield, 1971). All the subjects received an information sheet explaining the experimental procedures in detail. All of them reported no contraindications to TMS (Rossi et al., 2009, 2021), had normal or corrected to normal visual acuity. HS provided a written informed consent in which they also declared to have no history of neurological, psychiatric, including current or previous mood conditions, chronic pain syndrome, history of previous major surgery in the head or neck area, history of seizures, heart pacemaker or electronic implant or other medical problems.

The study was conducted according to the principles expressed in the Declaration of Helsinki and was approved by the ethical committee of the Department of Neurological and Movement Sciences, University of Verona, Italy (prog. N°2899, approval number 48632, 14/09/2020). HS were companions of patients from our outpatient clinic, hospital staff and medical students. No financial compensation was provided for participation in the study.

### Electromyography Recording

Surface EMG was recorded from the motor point of the APB and ADM muscles with unipolar self-adhesive Ag-AgCl electrodes (1.5 × 2.5 cm) in a belly-tendon montage. The ground electrode was attached to the palm. EMG signals were amplified (1000×), filtered by bandpass from 10 to 1 kHz plus a notch set at 50 Hz filter (LabChart 8 pro, ADInstruments Co.), sampled at the frequency of 2 kHz and digitized by PowerLab 16/35 (ADInstruments Co.). EMG signals were analyzed in real-time and offline using MATLAB (MATLAB 2014a, MathWorks Inc.). At the selected time intervals, the trigger signals were sent automatically from MATLAB to the TMS machine using a USB-6009-NI (National Instruments Corporation).

### Transcranial Magnetic Stimulation

Focal TMS was applied over the M1 of the dominant side (DS) and non-dominant side (NDS) through a standard figure-of-eight coil with mean loop diameters of 9 cm connected to a Magstim^2^ Rapid stimulator (The Magstim Company, Whitland, United Kingdom), randomizing the starting session order. The coil was mounted on an articulated arm and positioned tangentially to the skull at an angle of 45° to the sagittal plane (Emadi Andani et al., 2015). MEP_APB_ amplitude was the primary dependent measure in this study. The ADM is a task-irrelevant muscle and was used as a control muscle for the assessment of MEP changes: it was activated by the TMS but, unlike the APB, it was not directly involved in the task (Duque et al., 2010). The APB optimal scalp position was identified by moving the coil in small steps laterally to vertex in the hemispheres and by delivering TMS pulses with constant intensity until stable and maximal MEP could be evoked in the relaxed APB muscle. After having identified the hot spot for APB, the coil position was recorded using the SofTaxic Neuronavigation System (The E.M.S. srl Co.) that combines MRI-based image guidance with non-invasive TMS. The SofTaxic System consists of the SofTaxic Neuronavigation Software equipped with an optical digitizer (NDI Polaris Vicra). The system uses stereotactic localization of the TMS coil to visualize interactively the calculated spot of the stimulation and to guide the TMS precisely over the cortical areas of interest. The RMT was defined as the lowest stimulus intensity able to evoke MEP with an amplitude of at least 50 μV in at least five out of ten trials in the APB muscle. High gain visual EMG monitoring was used to ensure complete muscular relaxation. Pre-stimulation EMG level was evaluated by calculating the root mean square of the background EMG activity over 50 ms prior to the MEP onset. Trials in which the MEP_APB_ amplitude was lower than the mean background EMG activity were removed (Alaerts et al., 2010). All the trials in which the root mean square of the background EMG activity of the APB and ADM muscles was >10 μV were removed (Coxon et al., 2006). Moreover, all the neurophysiological data were inspected to rule out outliers (i.e., values 2.5 × SD above or below the mean value for each subject in each session). MEP amplitude from the APB and ADM were also recorded with subjects at rest (MEP_REST_), by stimulating at 120% RMT.

### Pre-movement Facilitation

We assessed PMF during a simple RT motor task (Chen et al., 1998; Morgante et al., 2011). During the experiment subjects were comfortably seated in a chair in front of a computer monitor (29 cm × 38.6 cm) with their arm slightly abducted from the trunk by about 45°–50°, flexed at the elbow at ∼90° with the forearms resting on a table to ensure complete arm relaxation. All participants had normal or corrected to normal visual acuity.

**FIGURE 1:**
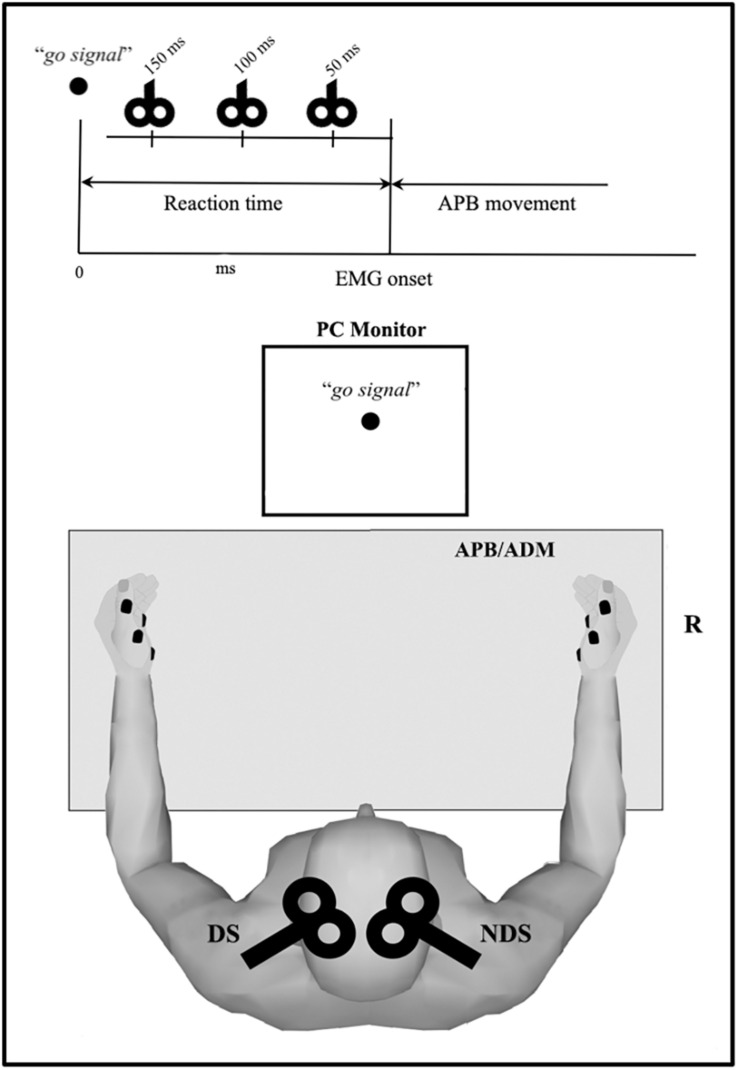
Overview of the reaction time (RT) protocol. Motor task consists of rapid thumb abduction; transcranial magnetic stimulation (TMS) was delivered after the visual “go signal” and before the electromyography (EMG) onset (i.e., within the RT) at three intervals (50, 100, and 150 ms). Motor evoked potential were recorded from APB and ADM, bilaterally. APB, abductor pollicis brevis; ADM, abductor digiti minimi.

The experimental session consisted of three parts, repeated for each side: we first assessed for each subject the mean RT by recording 10 trials in which the subject briskly abducted the right thumb in response to a visual go signal (black circle, diameter: 4 cm) in order to produce a single EMG burst, with the hand. Before recordings, each subject had one practice trial to ensure familiarity with the task. Subjects were asked to maintain complete relaxation between trials and all of them were able to perform properly the task after few minutes training. Second, we recorded 10 control TMS trials to assess the mean amplitude of the control MEP_REST_ (baseline condition). Finally, we recorded a block of 30 RT trials combined with TMS delivered after the go signal and before EMG onset at 150 (TMS_150_), 100 (TMS_100_), and 50 ms (TMS_50_) (ten trials each) (Figure 1), calculating the mean RT for each TMS timing (RT_150_, RT_100_, and RT_50_). The order of RT-TMS trials at different time intervals was randomly intermixed. The trials were repeated every 8 ± 1 s. The peak to peak MEP amplitude at 150 (MEP_150_), 100 (MEP_100_), and 50 ms (MEP_50_) before EMG burst was evaluated and then compared to the MEP_REST_.

All the experimental procedures were performed bilaterally, randomizing the starting session order.

### Statistical Analysis

Results were expressed as mean ± standard deviation. Data were assessed for normality using the Shapiro-Wilk test. A 2-way rmANOVA SIDE (dominant, non-dominant) × TIME (rest, 50, 100, and 150 ms) was performed to compare RT across conditions. A 3-way rmANOVA (TIME × SIDE × MUSCLE) was performed to test for changes in MEP amplitude. *Post hoc* comparisons were run in the case of finding significant effects. Alpha *p*-levels obtained from paired comparisons between rest and the other TMS conditions were Bonferroni-corrected.

## Results

**FIGURE 2:**
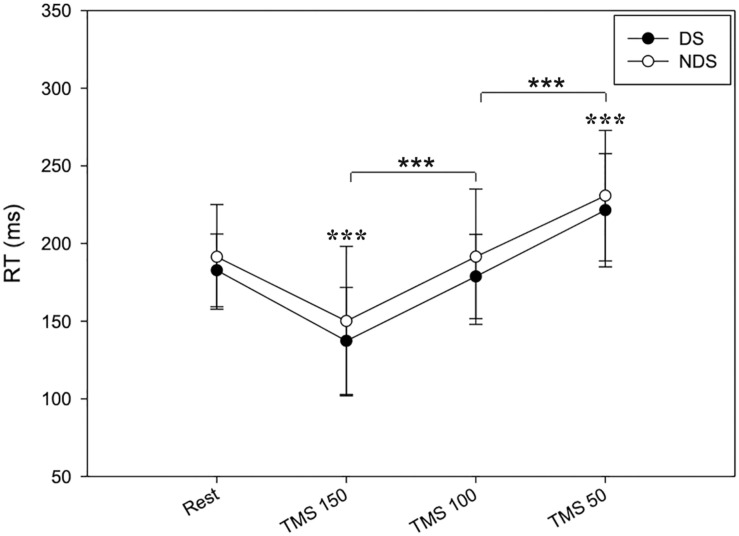
Timing of RT changes at rest and after TMS. RT reduces when TMS was delivered at 150 ms and progressively increases when TMS was delivered at 100 and 50 ms before the EMG onset. No significant changes were detected between the two sides. Data expressed as mean ± standard deviation (rmANOVA ***p < 0.001).

Tables 1–3 provide a summary of the neurophysiological findings. There was no change in the RMT measured in the DS (75.40 ± 10.81) and NDS (75.73 ± 9.08) (*p* = 0.906). RT_REST_ and during all the timings of the TMS protocol (RT_150_, RT_100_, and RT_50_) was slightly, though not significantly, shorter in the DS when compared to the NDS (Table 1 and Figure 2). Figure 2 plots the average RT in TMS trials compared with baseline: if the TMS pulse was delivered within the RT, 150 ms before onset of EMG activity, movement start was anticipated; in contrast, movement onset was delayed if TMS pulses were given 100 or 50 ms before EMG onset (see Table 1) (rmANOVA “time and side”, *F* = 3.72, *p* < 0.001), highlighting the time-dependent effects of TMS on motor facilitation.

**TABLE 1:** Reaction time (RT) at rest and after transcranial magnetic stimulation (TMS).

	RT (ms)		*p* value		
Time	DS	NDS	DS vs. NDS	vs. bas. DS	vs. bas. NDS
RT_REST_	182.6 ± 22.4	191.3 ± 33.7	0.185	/	/
RT_150_	137.2 ± 34.5	150.1 ± 48.1	0.286	**<0.001**	**0.001**
RT_100_	178.7 ± 27.1	191.4 ± 43.5	0.614	0.583	0.655
RT_50_	221.4 ± 36.4	230.7 ± 42.1	0.548	**<0.001**	**<0.001**

*RT, reaction time; ms, millisecond; DS, dominant side; NDS, non-dominant side; bas., baseline. Data expressed as mean ± st.dev. Bold values indicate the statistically significant data.*

**TABLE 2:** Motor evoked potential amplitude at rest and after TMS.

		MEP amp. (mV)		*p* value		
Muscle	Time	DS	NDS	DS vs. NDS	vs. bas. DS	vs. bas. NDS
APB	MEP_REST_	0.77 ± 0.18	0.73 ± 0.09	0.901	/	/
	MEP_150_	1.62 ± 0.30	1.03 ± 0.19	0.301	**0.0192**	**0.00449**
	MEP_100_	1.65 ± 0.31	1.27 ± 0.23	0.106	**0.018**	**0.00072**
	MEP_50_	2.45 ± 0.30	2.33 ± 0.46	0.947	**0.000021**	**0.00015**
ADM	MEP_REST_	0.79 ± 0.59	0.68 ± 0.43	0.412	/	/
	MEP_150_	0.58 ± 0.33	0.32 ± 0.17	0.580	0.752	0.732
	MEP_100_	0.53 ± 0.28	0.36 ± 0.22	0.749	0.692	0.653
	MEP_50_	0.56 ± 0.27	0.42 ± 0.21	0.806	0.716	0.497

*MEP, motor evoked potential; amp., amplitude; APB, abductor pollicis brevis; ADM, abductor digiti minimi; DS, dominant side; NDS, non-dominant side; bas., baseline. Data expressed as mean ± st.dev. Bold values indicate the statistically significant data.*

**TABLE 3:** Background EMG activity at rest and after TMS.

		BKG-EMG activity		*p* value		
Muscle	Time	DS (μV)	NDS (μV)	DS vs. NDS	vs. bas. DS	vs. bas. NDS
APB	Rest	24.96 ± 3.19	18.26 ± 5.10	0.911	/	/
	TMS_150_	27.27 ± 5.28	20.96 ± 3.01	0.065	0.114	0.243
	TMS_100_	30.42 ± 4.89	23.29 ± 3.28	0.228	0.167	0.13
	TMS_50_	30.65 ± 5.65	27.51 ± 7.55	0.741	0.463	0.159
ADM	Rest	26.76 ± 8.70	20.59 ± 10.57	0.787	/	/
	TMS_150_	23.70 ± 4.88	17.83 ± 4.37	0.384	0.764	0.283
	TMS_100_	24.81 ± 5.28	19.99 ± 5.29	0.532	0.85	0.384
	TMS_50_	27.54 ± 5.83	27.47 ± 10.09	0.995	0.941	0.833

*BKG-EMG, background electromyographic activity; APB, abductor pollicis brevis; ADM, abductor digiti minimi; DS, dominant side; NDS, non-dominant side; bas., baseline. Data expressed as mean ± st.dev.*

**FIGURE 3:**
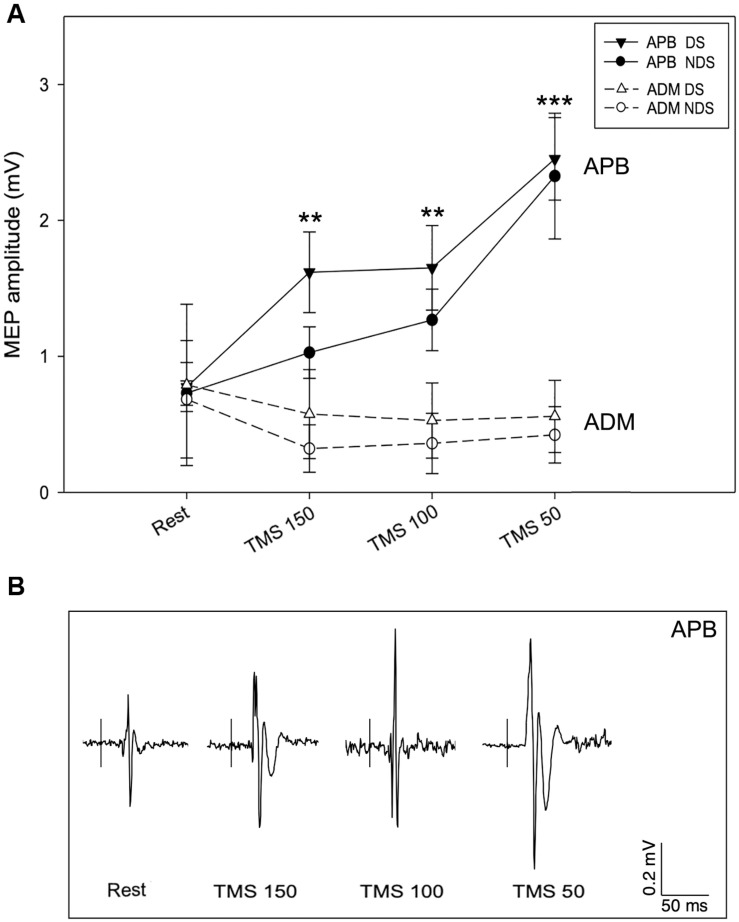
Motor evoked potential amplitude at rest and after TMS. **(A)** In APB, TMS increases MEP amplitude when delivered 150 (MEP150), 100 (MEP100), and 50 ms (MEP50) before the EMG onset, suggesting the occurrence of premovement facilitation (PMF; note the highest increase of MEP50). No significant changes of MEP amplitude were recorded from ADM, bilaterally. **(B)** Representative MEPs from APB in the different experimental conditions. Data expressed as mean ± standard deviation (rmANOVA, **p < 0.05; ***p < 0.001). APB, abductor pollicis brevis; ADM, abductor digiti minimi; MEP, motor evoked potential.

We then analyzed the amplitude of MEP_REST_ and after the motor task with TMS provided at the three different intervals. No differences were detected for MEP_REST_ recordings from APB (*p* = 0.901) and ADM (*p* = 0.412), bilaterally. Again, comparing with MEP_REST_ recordings, there were no significant differences between DS and NDS but a significant amplitude increase of MEP_150_, MEP_100_ (^∗∗^*p* ≤ 0.05) and MEP_50_ (^∗∗∗^*p* ≤ 0.001) (Figure 3A and Table 2) (rmANOVA DS: *F* = 5.8; *p* = 0.001; NDS: *F* = 4.87; *p* = 0.003), confirming the occurrence of PMF. Representative MEP from a HS are shown in Figure 3B. Interestingly, increase of MEP amplitude was greater in the DS compared to the NDS with TMS at 150 and 100 ms before EMG onset (rmANOVA “side × time”, *F* = 5.2, *p* = 0.025). This result suggests a different and early TMS-induced facilitatory effect on MEP amplitude on the DS.

The data analysis also demonstrates a selective PMF effect on the target muscle (APB) and not on ADM (task-irrelevant muscle). As presented in Table 2, in ADM we detected no changes of MEP_150_, MEP_100_, and MEP_50_ amplitude compared to the MEP_REST_ (Figure 3A) (rmANOVA “Time” × “Side” between sessions DS: *F* = 0.09; *p* = 0.96; NDS: *F* = 0.34; *p* = 0.79), thus demonstrating a selective PMF effect on the active muscle.

**FIGURE 4:**
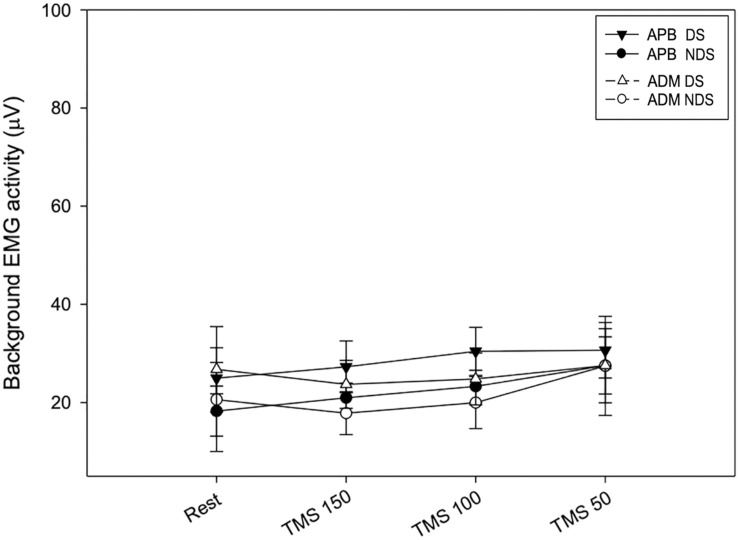
Background EMG activity at rest and after TMS. No changes were reported at rest and after TMS at the three intervals. Data expressed as mean ± standard deviation. APB, abductor pollicis brevis; ADM, abductor digiti minimi.

Finally, to verify whether the MEP amplitude was influenced by the preceding EMG activity, we analyzed the background EMG activity before the MEP onset for both APB and ADM (Figure 4). Our analysis disclosed no significant effects of the factors “Time” and “Side” or their interaction between sessions (APB DS: *F* = 3.62; *p* = 0.15; NDS, *F* = 0.47, *p* = 0.94; ADM DS, *F* = 0.07, *p* = 0.97; NDS: *F* = 0.56; *p* = 0.64), suggesting that background EMG activity was similar in all the sessions, thus the MEP amplitude was not influenced by differences in the preceding EMG activity.

## Discussion

We here have reported a selective and facilitatory effect of corticospinal excitability in the preparation phase of simple voluntary movements, as assessed by single pulse TMS on M1 during a simple RT protocol. MEP amplitude increased to a higher extent when TMS was delivered within the RT, shortly before (50 ms) before EMG onset. Moreover, we observed a TMS time-dependent change of RT during the protocol and a greater effect of TMS on the MEP amplitude when stimulated the DS compared to the NDS.

Premovement facilitation is defined as an increase of MEP amplitude/probability of MEP appearance after the *go signal* and before movement onset and begins approximately 100 ms before the EMG/movement onset (Rossini et al., 1988; Hallett et al., 1991; Pascual-Leone et al., 1994a; Chen et al., 2001; Hiraoka and Abe, 2010; Hiraoka et al., 2010a). Before movement onset, activation of motor neurons in M1 reflects the premovement increase of cortical excitability. This movement-related cortical potential (MRCP), which reflects the cortical functions underling voluntary movements, begins about 1–2 s before movement and might be discomposed into three main components (Lattari et al., 2014): (i) the *Bereitschaftspotential* (BS), also called readiness potential, reflects the cerebral activity prior to a motor response; (ii) the *Negative Slope Potential* (NSP), a rapid negative shift observed 500–600 ms before the movement/EMG onset; and (iii) a *Movement Potential* (MP) occurring 50–100 ms before the EMG onset that reflects the excitability of pyramidal cells in M1 (Chen et al., 1998; Hiraoka et al., 2010a). Accordingly, it was supposed that the timing of PMF eliciting an increase of corticospinal excitability for volitional movements is less than 100 ms and corresponds with the MP, thus confirming that PMF may be the result of the excitability of pyramidal cells in the M1 (Nomura et al., 2001). Our results are perfectly in line with these findings. The neural circuitries for movement execution share a common pathway with those involved in motor preparation, as demonstrated by kinesthetic motor imagery tasks (Decety, 1996; Jeannerod and Frak, 1999; Gerardin, 2000; Hanakawa et al., 2003; Vry et al., 2012). Accordingly, BS showed similar morphology and latency during motor imagery and execution tasks, however, the BS amplitude for motor execution is greater than that for motor imagery (Galdo-Álvarez and Carrillo-de-la-Peña, 2004; Carrillo-de-la-Peña et al., 2008).

Our data confirm previous results using simple movements compared to sequential movements protocols: the increase of MEP in the movement preparation phase, indeed, was greater as the onset of the MEP got closer to the movement onset (Rossini et al., 1988; Hallett et al., 1991; Pascual-Leone et al., 1994b; Chen et al., 2001). The greater PMF using sequential movement protocols suggests a recruitment of many other primary motor neurons, facilitation and a greater cortical efferent/corticospinal excitation for execution of complex movements (Flament et al., 1993; Tinazzi et al., 2003). Our protocol consisting of thumb abduction was preferred for selective contraction of the APB muscle, avoiding confounding co-contraction of adjacent muscles. Changes in the amplitude of H reflexes (Hayes and Clarke, 1978; Day et al., 1983; Eichenberger and Rüegg, 1984; Ruegg and Drews, 1991; Leocani et al., 2000) and in reciprocal inhibition (Day et al., 1983) have demonstrated facilitation of the efferent pathway to the target muscle during PMF. Non-invasive brain stimulation techniques, using both supra (TMS) and subthreshold stimuli (both TMS and electrical stimulation), have demonstrated a major probability to increase evoked motor responses in the target muscle beginning ∼100 ms before EMG onset (Rossini et al., 1988; Starr et al., 1988; Pascual-Leone et al., 1992; Hoshiyama et al., 1996, 1997; Chen et al., 1998; Leocani et al., 2000).

The importance of studying PMF has also been underlined in studies demonstrating an association between pathological fatigue, a frequent and disabling symptom common to many neurological diseases (Kluger et al., 2013; Di Vico et al., 2021) and lack of PMF patients affected by multiple sclerosis (Morgante et al., 2011). Moreover, reduced suppression and increased facilitation of corticospinal excitability prior to movement onset in post-stroke highly fatigued patients indicates poor modulation of pre-movement excitability which may in turn reflect poor sensory processing (De Doncker et al., 2021), supporting the sensory attenuation model of fatigue (Kuppuswamy, 2017). Importantly, these results may prompt to the hypothesis that reduced PMF is a reliable biomarker of fatigue in neurological illnesses and may reflect a dysfunction in motor planning and movement preparation in fatigued patients. Given the tremendous impact of fatigue on all shades of quality of life in neurological patients (Kluger et al., 2013), further research to improve our understanding of the exact implication of PMF in fatigue may be of high clinical value and it absolutely deserve increased attention. PMF was also found to be abnormal in patients with Parkinson’s disease, showing a direct and significant correlation with akinesia (Hiraoka et al., 2010b). Interestingly, also patients with spinocerebellar degeneration exhibited reduced PMF resulting in a longer latency in performing a given task and in a delayed reaction time (Nomura et al., 2001), thus underlying the complexity of movement preparation and execution and the role of other brain areas in both movement preparation and execution (Leuthold and Jentzsch, 2002; Errante and Fogassi, 2020).

We have also observed a greater effect on the MEP amplitude when TMS was delivered on M1 of the DS at 150 and 100 ms before movement onset. This asymmetrical and time-dependent MEP effects might result from the negative modulation of corticospinal excitability of the non-dominant brain hemisphere that favors the dominant when performing a movement with the dominant hand (Poole et al., 2018).

Our data also revealed that the TMS had distinct effects on the reaction times, thus affecting the timing of the volitional motor response. RT and movement onset were reduced by TMS given 150 ms before the average time of EMG onset and speeded by TMS delivered 100 ms and, predominantly, 50 ms before EMG onset. Previous studies have attributed this delayed effect to the silent period following the MEP, which suppresses EMG activity (Ziemann et al., 1997) and the speeded effect to the sensory input produced by the TMS pulse (the coil “click” and skin/muscle stimulation of the scalp), interpreted either as an intersensory facilitation (Nickerson, 1973; Ibáñez et al., 2020) or as a direct TMS effect on cortical processing, or both. Both the stimulus and sensory inputs from the TMS pulse are supposed to reduce the time for identification of the go-signal and speed up the EMG onset (Pascual-Leone et al., 1992; Leocani et al., 2000).

We have also reported a selective effect of TMS-PMF on the muscle involved in the task (APB), and not on the adjacent ADM muscle, in which we did not observed changes of MEP amplitude. We can deduce that ADM muscle does not participate in the task hypothesizing inhibitory mechanisms (Stinear and Byblow, 2003; Sohn and Hallett, 2004), restricted to the movement initiation phase and absent during tonic contraction (Beck et al., 2008). A difference between MEP in the two muscles was expected for two reasons: (1) the site of stimulation was over the APB “hotspot” and (2) the task-relevant muscle typically shows larger responses than the task-irrelevant one in RT task (Quoilin et al., 2019). Inhibitory mechanisms ensure selectivity of motor response through the surround inhibition that is the suppression of excitability in an area surrounding an activated neural network, in order to focus neuronal activity and to select neuronal responses (Beck et al., 2008). First described in the sensory/visual system for spatiotemporal discrimination of sensory inputs (Blakemore et al., 1970; Angelucci et al., 2002), surround inhibition is considered a relevant mechanism also in motor system allowing the selective movement execution (Ziemann et al., 1996; Sohn and Hallett, 2004; Beck and Hallett, 2011), counteracting the increased spinal excitability during movement initiation to preserve motor precision. Using TMS during motor activation protocols, it has been demonstrated that surround inhibition occurs earlier and to an higher amount with increasing motor task difficulty (Beck and Hallett, 2010) and active muscles show increased excitability while non-active muscles are inhibited (Sohn and Hallett, 2004; Shin et al., 2007). This selectivity is supposed to be achieved by intracortical inhibition of the area surrounding the cortical representation of muscles acting as agonist or synergist during task movement initiation, but not during the maintenance phase (Beck et al., 2008). Mechanisms of surrounding inhibition have not been fully clarified, however, motor excitability related to little finger movement is mainly suppressed at the supraspinal level through inhibitory mechanisms including those underlying the cortical silent period involved for silencing surrounding muscles (Sohn and Hallett, 2004).

Our study has a number of limitations worth nothing: first, we did not specifically investigate inhibitory circuits, however, our methodological setup and results demonstrate the main role of excitatory mechanisms and a selective PMF effect on APB. Second, MEP amplitude depends on the amount of corticospinal activity evoked by TMS as well on the excitability of downstream motor effectors, e.g., spinal motoneurons and interneurons. Therefore, it is important to acknowledge that MEP amplitude and the level of corticospinal excitability not only reflect the activity in the corticospinal pathway but also their modulation at the spinal level.

In conclusion, the current study supports the role of excitatory mechanisms of M1 (MEP increase) at the basis of the PMF during a RT motor task. The PMF effect was limited to the target muscle (APB) and not evident into ADM, a task-irrelevant muscle in which we did not observe changes in MEP amplitude, thus hypothesizing the role of intracortical or subcortical mechanisms of surrounding inhibition. Furthermore, we reported a side- and time-dependent increase of the MEP amplitude when TMS was delivered within the RT and a time-dependent modulation of the volitional motor response.

Future experiments are necessary to disentangle the mechanisms of PMF and to verify its role in the clinical context and in particular their role in neurological disorders.

## Data Availability Statement

The raw data supporting the conclusions of this article will be made available by the authors, without undue reservation.

## Ethics Statement

The studies involving human participants were reviewed and approved by University of Verona. The participants provided their written informed consent to participate in this study.

## Author Contributions

GC, IDV, ME, and GS: design of the study, data collection and analysis. GC, MB, and MT: interpretation of the data. GC: drafting the manuscript. GC, AT, FM, MB, and MT: critical revision of the manuscript. All authors contributed to the article and approved the submitted version.

## Conflict of Interest

The authors declare that the research was conducted in the absence of any commercial or financial relationships that could be construed as a potential conflict of interest.

## Abbreviations

MEP: motor evoked potential
TMS: transcranial magnetic stimulation
M1: primary motor cortex
PMF: premovement facilitation
RT: reaction time
APB: abductor pollicis brevis
ADM: abductor digiti minimi
EMG: electromyography
DS: dominant side
NDS: non-dominant side
HS: healthy subjects.
